# The feasibility of a training course for clubfoot treatment in Africa: A mixed methods study

**DOI:** 10.1371/journal.pone.0203564

**Published:** 2018-09-13

**Authors:** Tracey Smythe, Rosalind Owen, Grace Le, Esperance Uwizeye, Linda Hansen, Christopher Lavy

**Affiliations:** 1 International Centre for Evidence in Disability, London School of Hygiene & Tropical Medicine, London, United Kingdom; 2 Global Clubfoot Initiative, London, United Kingdom; 3 Nuffield Department of Orthopaedics, Rheumatology and Musculoskeletal Sciences, University of Oxford, Oxford, United Kingdom; 4 CURE International, Kigali, Rwanda; 5 CURE International, Beit CURE Hospital, Lusaka, Zambia; Indiana University, UNITED STATES

## Abstract

**Background:**

There is no available training programme with standard elements for health workers treating clubfoot in Africa. Standardised training with continued mentorship has the potential to improve management of clubfoot. We aimed to evaluate the feasibility of such a training programme among clubfoot providers in Africa, and assess implications for training effectiveness and scale up.

**Method:**

We used participatory research with trainers from 18 countries in Africa over two years to devise, pilot and refine a 2-day basic and a 2-day advanced clubfoot treatment course. (The Africa Clubfoot Training or ‘ACT’ Course.) The pilots involved training 113 participants. Mixed methods (both qualitative and quantitative) were used for evaluation. We describe and synthesise the results using the eight elements proposed by Bowen et al (2010) to assess feasibility. All participants completed feedback questionnaires, and interviews were conducted with a subset of participants. We undertook a narrative description of themes raised in the participant questionnaires and interviews. Descriptive statistics were used to compare pre- and post-course scores for confidence and knowledge.

**Results:**

113 participants completed pre and post-course measures (response rate = 100%). Mean participant confidence increased from 64% (95%CI: 59–69%) to 88% (95%CI: 86–91%) post course. Mean participant knowledge increased from 55% (95%CI: 51–60%) to 78% (95%CI: 76–81%) post course. No difference was found in mean for either subscale of cadre or sex. The qualitative analysis generated themes under four domains: ‘practical learning in groups’, ‘interactive learning’, ‘relationship with the trainer’ and ‘ongoing supervision and mentorship’

**Conclusion:**

The Africa Clubfoot Training package to teach health care workers to manage clubfoot is likely to be feasible in Africa. Future work should evaluate its impact on short and long term treatment outcomes and a process evaluation of implementation is required.

## Introduction

Clubfoot is a common congenital disorder that causes mobility impairment if untreated. The Ponseti method for clubfoot management is recommended for the correction of the fixed foot deformity [1] and can be effectively delivered by mid-level personnel [2]. The treatment consists of sequential correction of the deformity through manipulation and casting, with a tenotomy in most cases, followed by the use of a foot abduction brace to maintain correction [3]. To date, no structured training programme with standard elements exists in Africa.

General strategies to improve clinical practice include didactic teaching, printed educational materials, audit and feedback, interactive workshops, use of local opinion leaders and computerised decision support systems. The effects of these interventions vary from minor to moderately large [4]. There is limited exploration of the development of training for clubfoot providers in the literature, with existing research largely restricted to outcomes of treatment. Evidence suggests that multifaceted training strategies have a higher efficacy, particularly when tailored to address specific barriers and settings [5] and the development of the Africa Clubfoot Training (ACT) project was therefore underpinned by adult learning theory, which is essentially experiential [6, 7].

Our preliminary research suggested that the training developed for clubfoot treatment providers should include a more basic and repetitive introductory element, understandable learning outcomes, practical skill development and elements to improve problem solving [8]. Pre-existing training materials were deemed by users to be too complex for mid-level learners and to contain an excess of information. The materials were thought to be confusing in the way that complex clinical theories (such as kinematic coupling) were presented to participants with a lower level of anatomical knowledge, and to provide insufficient links between assessment and treatment. The challenges of teaching mixed cadres of clinicians were noted, as well as the need for a clearly structured learning experience. The resulting ACT course therefore included two-day basic and advanced training courses, which consist of large group presentations, small group discussions, practical exercises and problem solving scenarios, and a training of trainers course. The content of the basic and the advanced course is included in Supplementary Information files (S1 and S2 Tables respectively). Materials included training manuals, presentation slides, videos, practical exercises and guidelines for supervision and mentoring.

The feasibility of such training has not previously been reported. Understanding the feasibility of delivering the training, and the experiences of participants is crucial for the intervention to be effective beyond the pilot phase. To address this gap, we aimed to evaluate the development of the ACT course.

## Method

### Study design

We conducted a mixed methods evaluation between March 2015 and February 2017, based on a model proposed by Bowen et al (2010). The model is designed to assess the feasibility of public health interventions [9] and we evaluated the training from the eight facets of acceptability, demand, implementation, practicality, adaption, integration, expansion and limited efficacy. We evaluated both qualitative and quantitative data to gain a deeper understanding of the research findings.

### Setting

Participants were clubfoot treatment providers from Ethiopia, Rwanda, the UK and Kenya. Trainers were international experts and Anglophone and Francophone regional trainers from throughout Africa. The training materials were appraised on nine occasions. The Basic Provider Course (BPC) was trialled four times (Ethiopia x3, Rwanda x1) and the Advanced Provider Course (APC) was trialled five times (Ethiopia x2, UK x1, Kenya x2).

### Recruitment

All participants were sent information about the training prior to the start. There were no exclusion criteria and we invited all participants attending the training to participate in the evaluation.

### Data collection

The extensive needs analysis and project planning, including the project funding application and minutes from the project team meetings, provided contextual background to inform our training design and application. The needs analysis included mapping of existing clubfoot service delivery and training opportunities, and information collected from key stakeholders including national and regional clubfoot programme directors, existing trainers and clubfoot service providers, and discussion with parents.

Semi-structured interviews with key informants and trainers started prior to the first training and continued throughout all nine training courses. The interview schedule was subdivided into three parts:

1. Questions concerning the process and structure of the training, to understand the demand for and acceptability of the training;
2. General questions about practical training opportunities to identify relevant activities to implement;
3. Specific questions about gaining knowledge and skills, to inform how to integrate mentoring.

Participants completed paper questionnaires by hand pre- and post-training. The key sections in the questionnaires included: participant demographics, the ranking of seven aspects of the course from most to least important, the most useful thing that was learned on the course and how to improve the materials. The questionnaires also included pre- and post-training confidence and knowledge on management of clubfoot. The confidence question format was statements to be scored on a five point Likert scale, ranging from “not at all confident” to “completely confident”, and the knowledge section was a single correct answer multiple choice question. A repeated-measures design examined changes immediately after the training session. Satisfaction questions were included on the end line questionnaire (S3 Table).

National clubfoot supervisors completed a paper based skills checklist (S4 Table) in routine supervision visits to the participant’s clinic within three months of completing the course (Ethiopia and Rwanda).

A six-month follow up electronic survey was sent to all regional trainers who participated in the ACT courses held in January and July 2016 to understand uptake.

### Data management and analysis

Two researchers in Public Health collated the responses to the open-ended questions into a word document and undertook a qualitative content analysis using the questions as the codes; similar responses were grouped, groups were titled and the number of responses counted. Using conventional content analysis, coding groups were derived directly from the text data [10]. We analysed satisfaction questions thematically and undertook a narrative description of themes raised in trainer questionnaires, interviews and the electronic survey. As the pilot training courses were conducted over a number of months, with the materials being revised after each pilot, the themes were examined for evidence of change over time in the participants’ perceptions of the training.

All quantitative data were entered into a Microsoft Excel 2000 (Microsoft Inc., Redmond, Washington) software package and managed and analysed data using Stata 14.2 (StataCorp 4905, Lakeway Drive College Station, Texas 77845, USA). Categorical and nominal/ordinal data were analysed using simple descriptive statistics, including calculation of proportions, and exploration of a comparative analysis of cadre (background of participant) and sex. For the composite confidence score, all participants’ Likert scale scores were added together, and the means and standard deviations (SD) were calculated.

Detailed descriptive statistics for the participants are provided in our research that reports on the development of the training course [8] and included in S1 Data: descriptive statistics of participants.

### Ethics

This study was approved by the Ethics Committee of the London School of Hygiene & Tropical Medicine, ref:10412 /RR/3466. Informed written consent was obtained from all participants.

## Results

### Acceptability

The content of the training materials and style of learning were found to be effective and appreciated by participants.

‘*this was exactly the material that I needed to advance my knowledge at this stage*’.

(Participant, Advanced Course)

Participant satisfaction improved when the limitations and parameters of the course were explained clearly in advance, as this allowed participants to manage their own expectations of the course.

Initial discussions on content resulted in debates on some areas, for example, on how much to correct a clubfoot before scoring its severity, about which there is little in the published literature. Contentious points were recorded and discussed with all stakeholders and consensus gained on presentation in the training [8]. In the final pilot courses, these consensus points were presented without generating much discussion, indicating their acceptability to the users.

The programme deliverers determined the training to be suitable through direct observation of engaged participants; supervision was provided by trainers (ratio of 1 trainer to 4 participants), which assisted the consistent engagement of all participants. The training approaches were found to be highly acceptable to participants, with several commenting that they appreciated the time for building relationships with the trainers and the informal learning and mentoring that occurred during the course.

The opportunity for hands on practical training was valued:

‘*Faculty with good physical dexterity, explained well positioning of the thumb, index and middle finger while correcting*.’

Participant casting on model legs

However, in the final versions of the training, when timing that allowed for practical sessions was maximised, requests from participants to have more hands on practice remained.

Some practical sessions included treatment of children with clubfoot. The factors that reduced acceptability for families included: insufficient explanation to parents, crowding of participants, taking photographs without permission, and parents’ embarrassment in being questioned in front of a large group. Clear guidance to manage these situations was subsequently developed in response.

### Demand

The needs analysis and pre-ACT consultation with existing trainers revealed a high level of demand for the development of a standardised training course. Emphasis on supervision and mentoring was built into all components of the training course, as identified in the needs analysis and requested by users. The majority of participants requested follow up training:

“*very useful and will definitely help us*…*repeat training is necessary*”

(Participant quotation).

Participants requested that a training structure be developed in their country so that regular refresher training and advanced training is available, and that their colleagues attend future courses.

### Implementation

Trainers reported the challenge of presenting materials that they themselves had not written, and the need for very clear guidance around the topics to cover when delivering each section of the training, the timings of each component and the instructions for interactive and practical sessions. As this guidance was refined, trainers’ satisfaction with the materials improved. Participants noted that the training delivery was aided with an identified course leader to direct each course; specific guidance for course leaders was developed and included in the training materials, and mentoring by more experienced trainers was given to new course leaders of ACT courses, to develop local course leadership capacity.

### Practicality

Environmental considerations for the training include adequate space, audio-visual equipment, ventilation, accommodation and refreshments. Participants reported that these issues played a very important role in the learning experience overall. Much organisation and support of local administration staff was required to identify appropriate participants and children with clubfoot and to assist in the daily running of the training course, the implementation of which was not assessed outside of the nine pilots. In addition, the increased volume of children for the clubfoot programmes after the training required consideration of local staffing requirements to absorb additional cases.

### Adaption

The course materials were piloted in English in Ethiopia, Kenya and UK and in French in Rwanda, and provided many opportunities to consider both local contexts and their more global applications. After the initial pilots, trainers noted that some of the more active learning styles were less familiar to many local participants. The materials were designed to support an interactive and practical skills-based learning experience, including small group discussions, problem-based learning, case studies, teaching of practical skills, reflexive practice, action planning, using evaluation tools, and structured feedback on teaching, practical and communication skills. Ten clear and memorable key ‘take-home’ messages for the basic course were identified and agreed by the development team that were actively repeated and reinforced through the course. Trainers and participants reported enjoying using a variety of learning styles and finding these beneficial.

This training was provided to mixed groups of professionals and had the potential to lead to tensions due to medical hierarchies. Cadres of lead trainers were therefore mixed to model good relationships and communication, e.g. between physiotherapists and orthopaedic surgeons. In some cases, specific instructions were given to overcome professional boundaries that might affect treatment quality—e.g. any clinician assisting a tenotomy was encouraged by the surgeon trainer to prompt the surgeon if the tenotomy was not considered to be complete.

### Integration

16/35 (45.7%) of national trainers responded to the electronic survey sent at six months following attendance at either the January or July 2016 pilots. All (100%) of the respondents reported that they had been involved in training other healthcare workers in clubfoot treatment and used the materials provided. This included workshops, courses and lectures and the majority of trainers had trained between 16 and 25 providers in this time.13/16 (81%) of the trainers had been involved in mentoring other healthcare workers in clubfoot treatment since delivering the ACT course.

The respondents noted that the materials for the course facilitated change in the way that they teach, and the most commonly reported change involved the way of teaching a practical skill. All trainers wanted to integrate the ACT materials into their current training programme. Reasons given included the simplicity of the theoretical and practical material, that the materials underwent many reviews with stakeholders, that principles of adult learning are incorporated, and that the standardised approach is useful.

### Expansion

Training with the ACT materials occurred in 20 countries (Burkina Faso, Burundi, Cameroon, Congo-Brazzaville, DRC, Ethiopia, Ghana, Guinea Bissau, Kenya, Madagascar, Malawi, Niger, Rwanda, Senegal, South Africa, Tanzania, Togo, Uganda, Zambia, and Zimbabwe) between April 2017 and February 2018. The training augmented existing regional programmes in these countries and program co-ordinators were encouraged to formalise the continuing professional development (CPD) gained through the ACT course on a local level. However, challenges include trainers not recruiting enough cases for the training, and requests to deliver the advanced course directly after the basic course with the same participants, as well as not understanding exactly what materials are needed to deliver the training.

### Limited efficacy

The BPC and APC training elicited change in the knowledge and confidence of the participants. Mean participant confidence increased from 64% (95%CI: 59–69%) to 88% (95%CI: 86–91%) post course. Mean participant knowledge increased from 55% (95%CI: 51–60%) to 78% (95%CI: 76–81%) post course (Table 1).

**Table 1:** Pre- and post-course self-reported confidence and knowledge performance by course.

Indicator	Course	Pre-course Mean % (95%CI)	Post-course Mean % (95%CI)
Self-reported confidence	TOTAL *	63.9 (58.9–69)	88.2 (85.9–90.5)
	BPC (n = 69)	59.7 (53.4–65.9)	89.1 (86.1–92.1)
	APC (n = 25)	77.0 (72.1–81.9)	86.2 (83.5–89.0)
Knowledge performance (single best answer multiple choice questionnaire)	TOTAL *	55.1 (50.7–59.5)	78.4 (75.8–80.9)
	BPC (n = 69)	53.9 (48.5–59.4)	80.3 (77.3–83.2)
	APC (n = 25)	58.8 (52.5–65.3)	72.5 (67.8–77.2)

* missing data due to incomplete questionnaires (n = 19)

**Fig 1:**
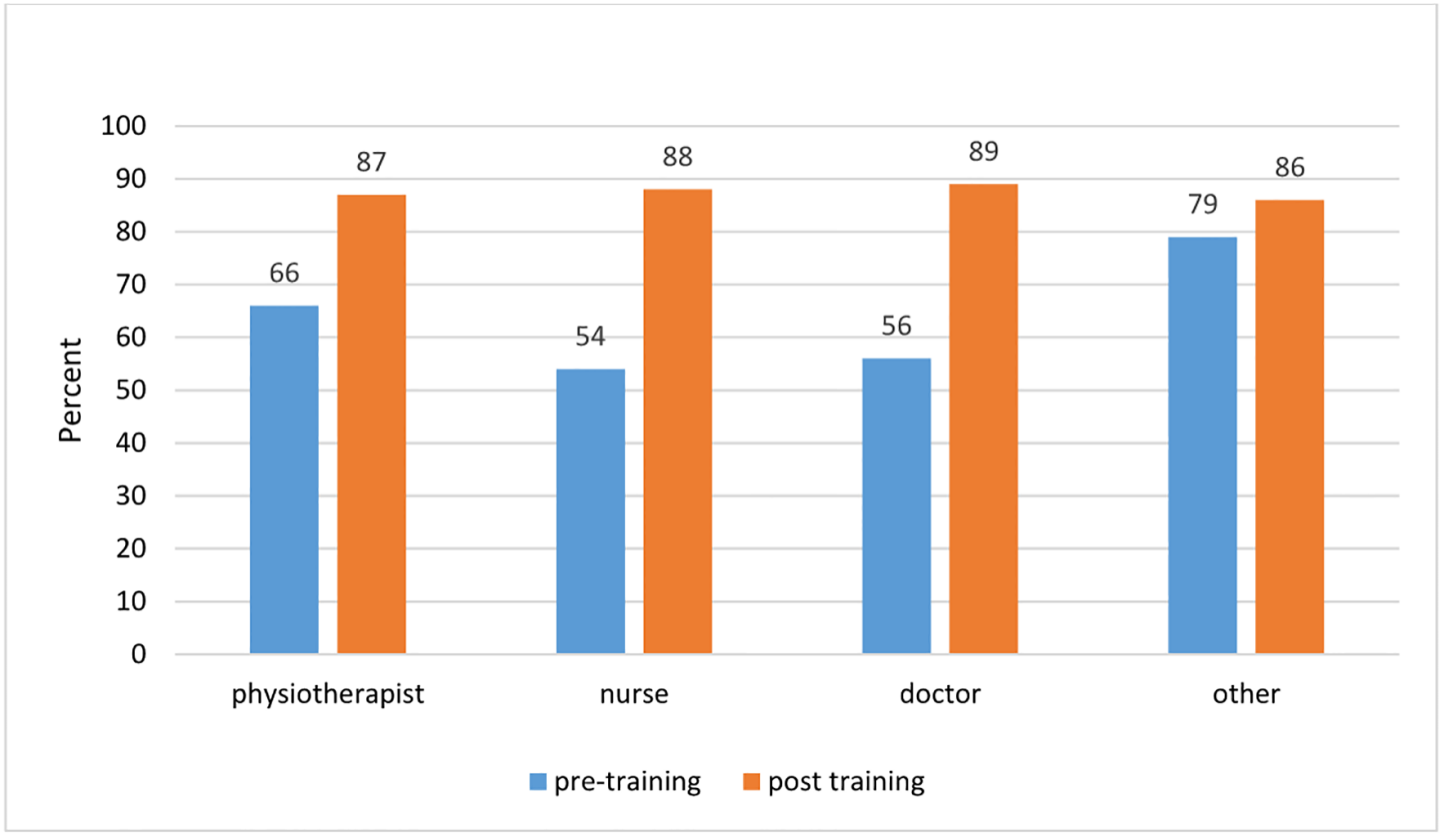
Pre- and post-course self-reported confidence as reported by cadre.

**Fig 2:**
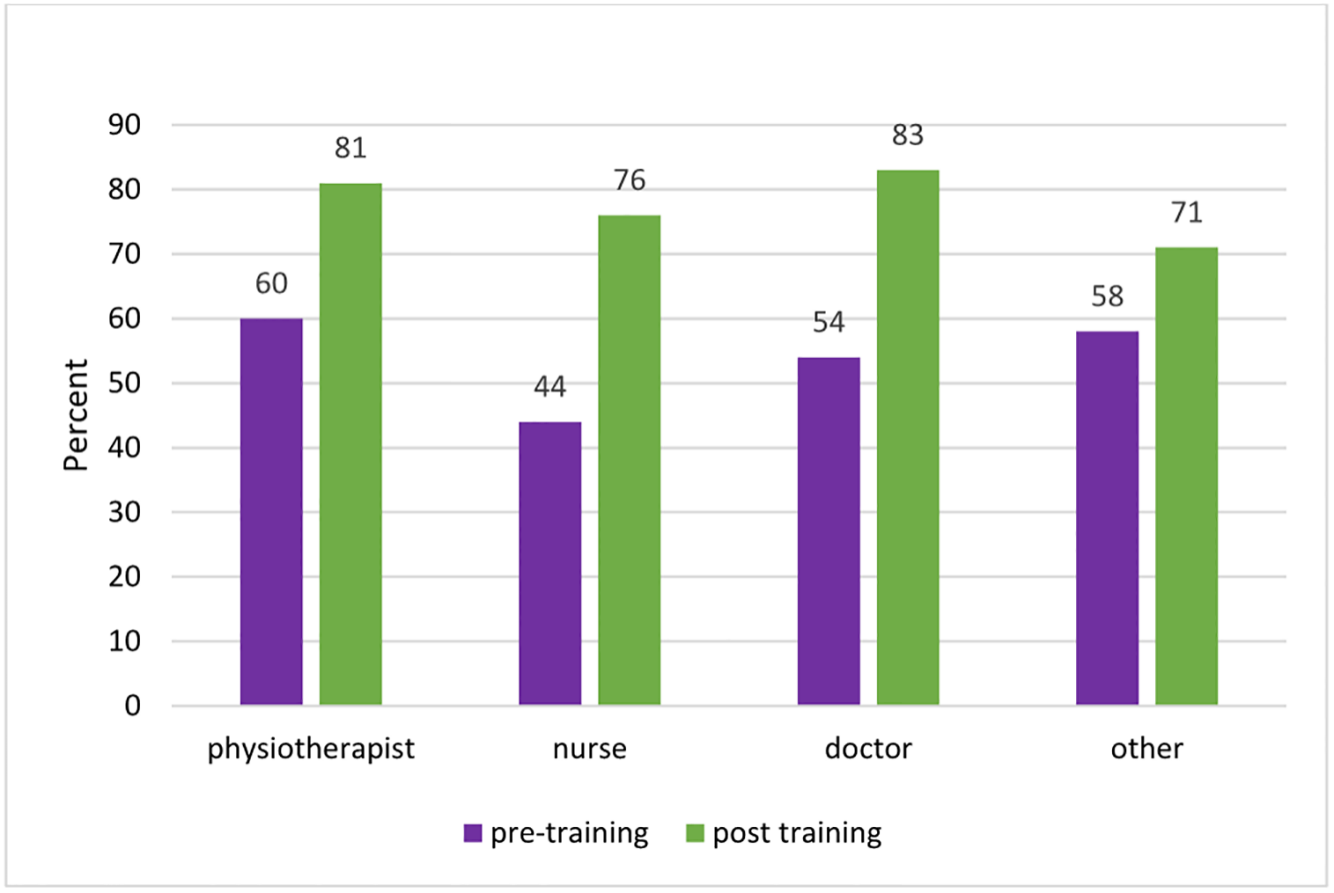
Pre- and post-course knowledge performance by cadre.

There was no evidence for a difference between sex in confidence or knowledge when cadre was accounted for. (Figs 1 and 2)

All participants practiced manipulation and casting on rubber models prior to the supervised treatment of children with clubfoot. The following comment reveals how a patient demonstration enabled a participant to improve their clinical reasoning skills:

*‘Live demonstration of the whole process from history taking to treatment was a good experience*…*has improved my professional judgement*.*’*

The ACT training may contribute to the successful supervised treatment of children with clubfoot (Ponseti method), even though the pilots were undertaken in a highly controlled setting.

Twenty-five participants who attended the clubfoot provider courses in Ethiopia were observed in their clinics. Over 80% (21/25) correctly assessed the clubfoot with the Pirani score and 92% (23/25) correctly manipulated the clubfoot. 22/23 health care workers met the criteria for correct application of braces. Participants met the fewest criteria in relation to discussing the Ponseti treatment with parents. Twenty participants who attended the clubfoot provider courses in Rwanda were observed in their clinics. Nineteen (95%) participants correctly assessed the clubfoot with the Pirani score and 17 (85%) correctly manipulated the clubfoot. 18 (90%) of the clubfoot providers met the criteria for correct application of braces. Supervisory notes identified one healthcare worker whom required additional support and assistance in several areas and a supervisory plan was initiated.

## Discussion

This study evaluates participant, trainer and stakeholder responses to the training of clubfoot providers through Africa. Participants expressed enthusiasm and a high acceptability of the program, which they attributed to its clear purpose and the interpersonal interaction with the trainers. The role of an available trainer in direct observation and mentoring of the participants to give feedback and support cannot be overemphasized. Success is further indicated through the request of tertiary institutes in the UK to use the training materials.

The development of the training material required substantial organisation and commitment to trial the facilitated participatory training programme nine times. The combined use of quantitative data and qualitative findings produced a course design that was feasible and acceptable. Participants appreciated the final clinical content for being clear and well structured. The basic course content was distilled, with clear key messages. The advanced course introduced more complex concepts, clinical reasoning and opportunity to reflect on one’s own practice.

The development of a skills checklist allowed the identification of opportunities to strengthen and improve clubfoot treatment in participants who attended clubfoot provider training. The breakdown of specific skills within the checklist allows for targeted mentoring. For example, although the overall casting of the child’s foot was performed well, one area that consistently required improvement was casting over the child’s toes. In Rwanda, a high percentage of health care workers achieved all the criteria (>80%). Supervising individuals with the skills list allowed for the identification of one participant whom required assistance and closer mentoring.

### Strengths and limitations

The methodology of this study allows a rich and deep understanding of the numerical data relating to the ACT, as well as the meaning that it had for both trainers and participants. This was a large prospective study that was situated in Anglophone and Francophone contexts. Study limitations include a short-term follow-up. The authors were closely involved in the implementation of the ACT project and in the development of the manuscript, which may lead to researcher bias. In addition, participants may have been reluctant to express their negative opinions or criticisms to the researchers since the interviewers were on the ACT development team. This study is not powered to detect a difference and there is no control group, however the figures for short-term improvement in knowledge and confidence indicate that this measure can be used in future training.

### Lessons learnt

Assessing our recruitment capability and resulting sample characteristics was important to determine if the training and future efficacy studies may be successful. Field notes and project team meeting notes were invaluable for the review of the content. Participants were eager to give feedback on the course materials and this provided an opportunity for ownership and engagement with the training course development. The selection of outcome measures was challenging and required continual assessment and re-evaluation. There was a need for clear instructions on how to run practical sessions in order to maximise their impact.

### Implications and future steps

An understanding of the barriers to the success of delivering the training within individual country contexts is required for implementation, and opportunities to enhance the technology used in the training warrant further examination. Evaluation of the effectiveness of national training is required in future studies, in addition to studies that explore the retention of knowledge and confidence post-training. As countries cascade the training, the numbers of participants that complete training will increase. Recognising the growing global collaboration to decrease clubfoot as a disability by 2030 [11] and the heterogeneity of measurement and assessment, perhaps the time has come to draft globally agreed minimum standards similar to those for medical education programmes.

## Conclusion

ACT has been received as a welcome addition to clubfoot treatment providers. Participants expressed high acceptability of the training, which they attributed to its clear purpose and guidance, convenience, and the interpersonal interaction with the trainers. The role of a supervisor, in direct observation of the participant, and dedicated mentoring that includes feedback and support, cannot be overemphasized. This is a particular challenge in low-resource countries due to the gap in services. The findings from this study are encouraging and merit further investigation in implementation process evaluations and larger clinical trials.

## Supporting information

S1 Table BPC timetable. (DOCX)

S2 Table APC timetable. (DOCX)

S3 Table Skills checklist. (DOCX)

S4 Table Pre-course confidence matrix. (DOCX)

S5 Table Demographics. (DOCX)

S1 Data Summary data of basic and advanced courses. (XLSX)
