# Supplementary material for: The feasibility of a training course for clubfoot treatment in Africa: A mixed methods study
Source: PLoS One. 2018 Sep 13;13(9):e0203564. doi: 10.1371/journal.pone.0203564 (PMC6136756; doi:10.1371/journal.pone.0203564)
Supplement: S1 Table — (DOCX) [file pone.0203564.s001.docx]

**S1: BPC Outline**

# Basic Clubfoot Treatment Provider Course (BPC) Timetable

| **Start** | **Mins** | **Number** | **Session** | **Trainer** |
| --- | --- | --- | --- | --- |
|  |  |  | **DAY 1** |  |
| 08:00 | 30” |  | Registration |  |
| 08:30 | 15” | 1 | Day 1 Opening Session (Welcome, course overview, introductions, ice-breaker, & formation of small groups) |  |
| 08:45 | 15” | 2 | Pre-course Assessment |  |
| 09:00 | 30” | 3 | Introduction to Clubfoot and the Ponseti Method |  |
| 09:30 | 30” | 4 | Anatomy and Definitions |  |
| 10:00 | 20” | 5 | The Clubfoot Deformity – CAVE |  |
| 10:20 | 30” |  | **Tea** |  |
| 10:50 | 30” | 6 | How to Assess the Severity of a Clubfoot Using the Pirani Score |  |
| 11:20 | 40” | 7 | The Ponseti Method of Manipulation |  |
| 12:00 | 30” | 8 | **Practical Session 1:**  Small group hands-on manipulation of rubber models and skeleton models |  |
| 12:30 | 45” |  | **Lunch** |  |
| 13:15 | 30” | 9 | Applying Clubfoot Casts |  |
| 13:45 | 45" | 10 | **Practical Session 2:**  Demonstration of Pirani score and casting (with patients) |  |
| 14:30 | 15” | 11 | Room Set up for Practical Session 3 |  |
| 14:45 | 30” |  | **Tea** |  |
| 15:15 | 90” | 12 | **Practical Session 3:**  Casting on rubber models (in small groups of 3) |  |
| 16:45 | 30” | 13 | **Practical Session 4:**  Ponseti video |  |
| 17:15 | 15” | 14 | Day 1 Closing Session |  |
|  |  |  | **DAY 2** |  |
| 08:30 | 20” | 15 | Day 2 Opening Session (Review) |  |
| 08:50 | 30” | 16 | The Tenotomy and When to Do it |  |
| 09:20 | 40” | 17 | Maintenance Phase: Bracing and Relapse |  |
| 10:00 | 30” |  | **Tea** |  |
| 10:30 | 150” | 18 | **Practical Session 5:**  Pirani score, manipulation, and casting with patients (in small groups of 3 or 4) |  |
| 13:00 | 60” |  | **Lunch** |  |
| 14:00 | 90” | 19 | **Practical Session 6:** (Parallel session)  Group 1: Brace fitting  Group 2: Tenotomy (theory & practical) |  |
| 15:30 | 30” |  | **Tea** |  |
| 16:00 | 30” | 20 | When to Stop and Rethink Treatment |  |
| 16:30 | 15” | 21 | Post-course Assessment |  |
| 16:45 | 15” | 22 | Day 2 Closing Session (Review, evaluation forms, and certificates) |  |
