# Supplementary material for: The feasibility of a training course for clubfoot treatment in Africa: A mixed methods study
Source: PLoS One. 2018 Sep 13;13(9):e0203564. doi: 10.1371/journal.pone.0203564 (PMC6136756; doi:10.1371/journal.pone.0203564)
Supplement: S2 Table — (DOCX) [file pone.0203564.s002.docx]

**S2: APC Outline**

# Advanced Non-Surgical Clubfoot Treatment Provider Course (APC) Timetable

| **Start** | **Mins** | **Number** | **Session** | **Trainer** |
| --- | --- | --- | --- | --- |
|  |  |  | **DAY 1** |  |
| 08:00 | 30” |  | Registration |  |
| 08:30 | 30” | 1 | Day 1 Opening Session (Welcome, course overview, & introductions) |  |
| 09:00 | 15” | 2 | Pre-course Assessment |  |
| 09:15 | 45” | 3 | A Review of the Ponseti Method for Idiopathic Clubfoot |  |
| 10:00 | 30” | 4 | Advanced Ponseti for Idiopathic Clubfoot |  |
| 10:30 | 30” |  | **Tea** |  |
| 11:00 | 45” | 5 | Common Errors in Clubfoot Management |  |
| 11:45 | 45” | 6 | Recognition and Treatment of Atypical Clubfoot (includes video) |  |
| 12:30 | 60” |  | **Lunch** |  |
| 13:30 | 45” | 7 | Recurrent Clubfoot |  |
| 14:15 | 45” | 8 | Treating Older Children |  |
| 15:00 | 30” |  | **Tea** |  |
| 15:30 | 90” | 9 | **Practical Session 1:**  1. Refresher of casting on rubber legs  2. Demonstration & practice of new handholds & atypical casting |  |
| 17:00 | 10” | 10 | Day 1 Closing Session |  |
|  |  |  | **DAY 2** |  |
| 08:30 | 15” | 11 | Day 2 Opening Session |  |
| 08:45 | 30” | 12 | Secondary Clubfoot |  |
| 09:15 | 15” | 13 | Briefing on Patients |  |
| 09:30 | 30” |  | **Tea** |  |
| 10:00 | 180” | 14 | **Practical Session 2:**  Case discussions (with assessment of patients, if available) |  |
| 13:00 | 60” |  | **Lunch** |  |
| 14:00 | 60” | 15 | Parent Education and Support |  |
| 15:00 | 60” | 16 | Clubfoot Clinic Set-up and Quality |  |
| 16:00 | 15” |  | **Tea** |  |
| 16:15 | 15” | 17 | Post-course Assessment |  |
| 16:30 | 30” | 18 | Day 2 Closing Session (Review, evaluation forms, and certificates) |  |
