# Supplementary figures and images for: The feasibility of a training course for clubfoot treatment in Africa: A mixed methods study
Source: PLoS One. 2018 Sep 13;13(9):e0203564. doi: 10.1371/journal.pone.0203564 (PMC6136756; doi:10.1371/journal.pone.0203564)

S4: ACT pre training confidence questionnaires for BPC


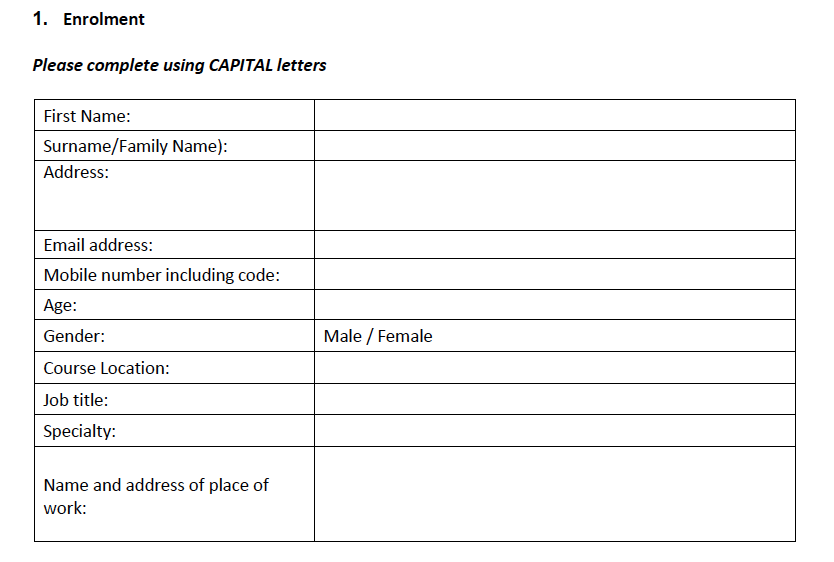


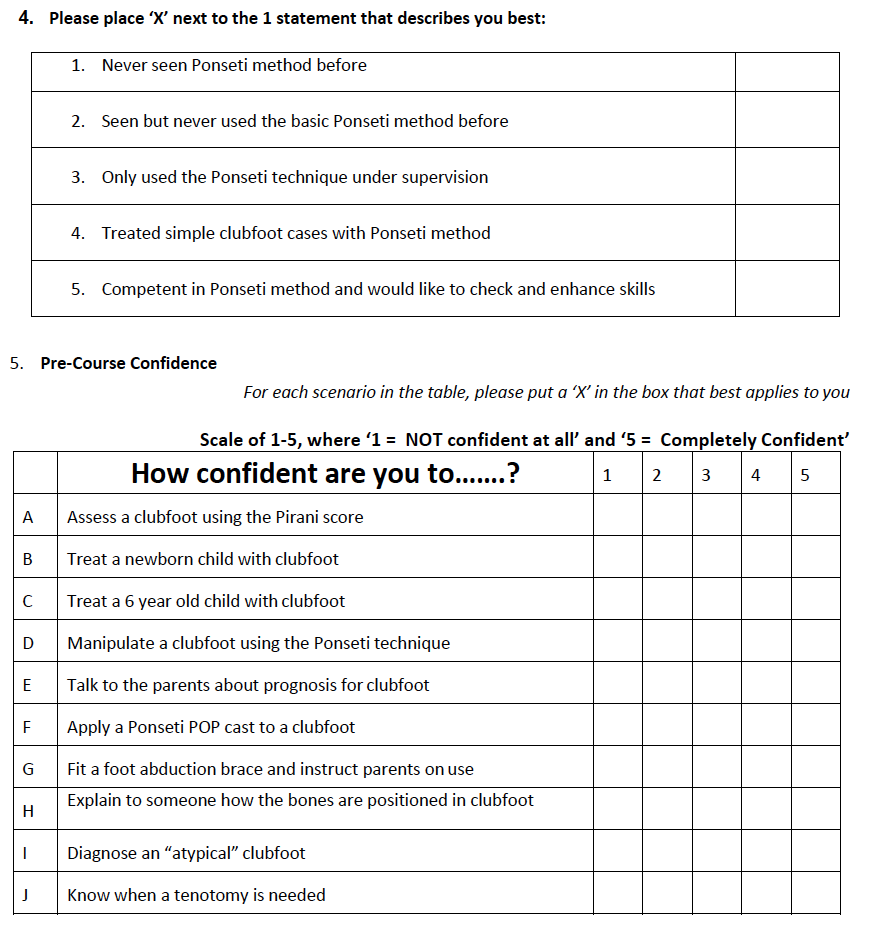


ACT post course confidence BPC


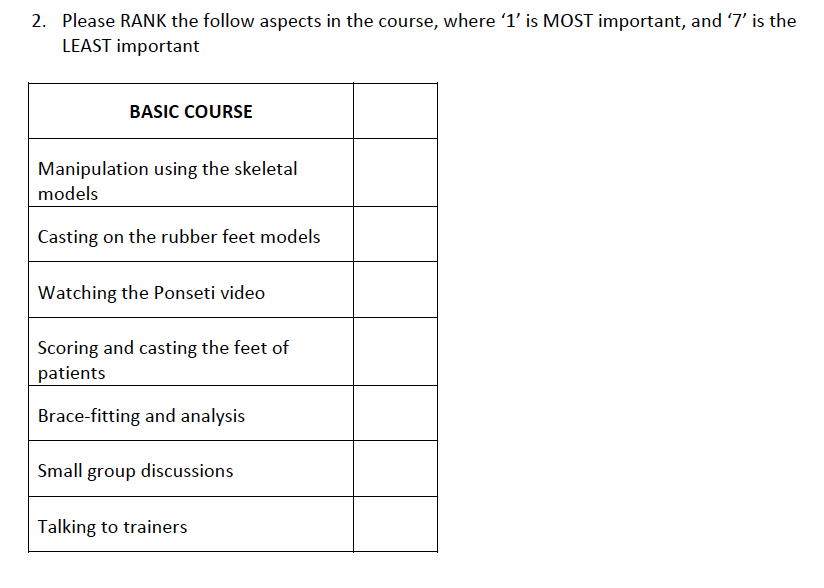


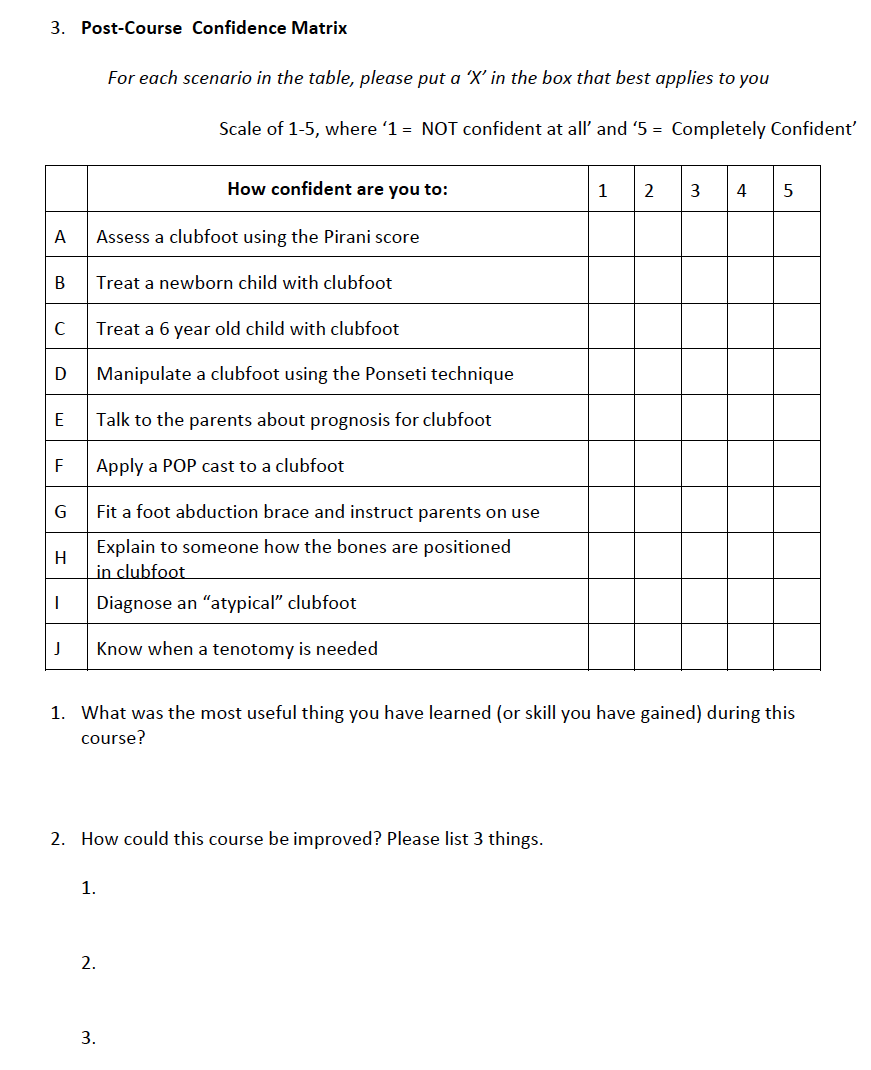

Supplement: S4 Table — (DOCX) [file pone.0203564.s004.docx]
