# Supplementary material for: The feasibility of a training course for clubfoot treatment in Africa: A mixed methods study
Source: PLoS One. 2018 Sep 13;13(9):e0203564. doi: 10.1371/journal.pone.0203564 (PMC6136756; doi:10.1371/journal.pone.0203564)
Supplement: S5 Table — (DOCX) [file pone.0203564.s005.docx]

**S5 Demographics of the pilot courses**

| Pilot number | 1 | 2 | 3 | 4 | 5 | 6 | 7 | Total** |
| --- | --- | --- | --- | --- | --- | --- | --- | --- |
| City  Country | Addis Ababa  Ethiopia | Addis Ababa  Ethiopia | Addis Ababa  Ethiopia | Kigali  Rwanda | London  (1 day) | Kenya (1day) | Kenya  (2 day) |  |
| Dates of pilot trainings | 23 – 24 September 2015 | 25 - 29 January 2016 | 25 - 29 July 2016 | 24 - 28 October 2016 | 2 December 2017 | 26 January 2017 | 24-25 January 2017 | 21 days |
| Type of pilot training | BPC and APC | BPC, APC and TTT | BPC and TTT | BPC and TTT | APC | APC | APC |  |
| Number of International experts delivering training | 8 | 9 | 9 | 9 | 5 | 5 | 5 | 21 |
| Countries represented by International Experts | Ethiopia, Norway, Netherlands, UK, Zimbabwe | Australia, Ethiopia, UK, Zimbabwe | Australia, Ethiopia, Kenya, Tanzania, UK, Zimbabwe | Australia, Canada, Cameroon, DRC, Rwanda, Switzerland, UK, Zimbabwe | UK | UK, Kenya, Australia | UK, Kenya, Australia | 13 |
| Number of organisers for training course | 4 | 3 | 3 | 3 | 2 | 3 | 3 | 12 |
| Countries represented (organisers) | Australia, Ethiopia, UK | Ethiopia, UK | Ethiopia, UK | Rwanda, UK | UK | Kenya, Zambia | Kenya, Zambia | 4 |
|  | | | | | | | | |
| Number of regional trainers | 0 | 18 | 17 | 16 | 0 | 1 | 1 | 51 |
| Number of countries represented (trainers) | Trainers were ACT faculty | 10 | 10 | 7 | 1 | 3 | 3 | 18 |
| Names of countries represented (regional trainers) |  | Ethiopia, Ghana, Kenya, Liberia, Malawi, Mozambique, Rwanda, Tanzania, Zambia, Zimbabwe | Ethiopia, Cameroon, DRC, Ghana, Kenya, Malawi, Sierra Leone, South Africa, Zambia, Zimbabwe | Burundi, Niger, DRC, Senegal, Cameroon, Rwanda, Togo |  |  |  | 18 |
| Cadres of trainers: surgeon | 5M | 11M | 9M | 5M | 0 | 0 | 0 | 30 |
| physiotherapist | 2F, 1M | 1F, 5M | 1F, 2M | 8M | 0 | 0 | 0 | 20 |
| medical doctor | 0 | 0 | 0 | 1F, 1M | 0 | 0 | 0 | 2 |
| clinical officer | 0 | 1 M | 3M | 0 | 0 | 0 | 0 | 4 |
| Nurse/other | 0 | 1F | 2M | 1M | 0 | 0 | 0 | 4 |
|  | | | | | | | | |
| Number of local providers trained | 20 | 17 | 18 | 21 | 12 | 12 | 13 | 113 |
| Cadres of providers trained: surgeon | 3M | 3M | 0 | 1M | 2M, 1F | 0 | 0 | 10 |
| physiotherapist | 1F, 7M | 1F, 3M | 1F, 9M | 3F, 9M | 2M, 6F | 1M, 2F | 2M, 1F | 48 |
| nurse | 7F | 4F, 5M | 2F, 1M | 2M | 1F | 1M | 0 | 23 |
| doctor | 2M | 1 M | 1F, 4M | 4M | 0 | 0 | 1M | 13 |
| other | 0 | 0 | 0 | 0 | 0 | 6M, 2F | 5M, 4F | 17 |
